# Supplementary material for: Lamellar projections in the endolymphatic sac act as a relief valve to regulate inner ear pressure
Source: eLife. 2018 Jun 19;7:e37131. doi: 10.7554/eLife.37131 (PMC6008045; doi:10.7554/eLife.37131)
Supplement: Transparent reporting form [file elife-37131-transrepform.docx]

***eLife’s* transparent reporting form**

We encourage authors to provide detailed information *within their submission* to facilitate the interpretation and replication of experiments. If you have any questions, please contact us: [editorial@elifesciences.org](mailto:editorial@elifesciences.org).

**Sample-size estimation**

- You should state whether an appropriate sample size was computed when the study was being designed
- You should state the statistical method of sample size computation and any required assumptions
- If no explicit power analysis was used, you should describe how you decided what sample (replicate) size (number) to use

Appropriate sample sizes were not computed when the study was being designed. The study began with an exploratory approach based on imaging and image analysis. The experimental approach and the manual image analysis are time consuming and labor intensive so we focused on aspects that could be measured and that were unmistakable such that several time courses would be sufficient to dismiss null hypotheses. The data presented in Figure 1G is dismissing two null hypotheses, that the endolymphatic sac does not inflate and that the time between peak inflations is a fixed period of time. In retrospect, the 8 time courses and 54 measurements were excessive for dismissing these two null hypotheses. Qualitatively they were necessary to saturate the distribution of time between peak inflations.

Appropriate sample sizes were not computed for the experiments presented in Figure 2. Figure 2B-D present a fluid phase tracer in the endolymph and qualitatively reinforce the observation in Figure 1. Figure 2E-F presents a qualitative finding that the ES deflates shortly after laser ablating cells in the otic vesicle. This experiment was done 4 times with time course evaluation and 7 times with endpoint analysis. Figure 2G present the qualitative finding that endolymph can leak out and perilymph can leak in through the course of a deflation. This experiment was done 8 times with time course evaluation.

Appropriate sample sizes were not computed for the experiments presented in Figure 3. Study of the mutant began in an effort to clone discarded ear morphogenesis mutants. While cloning the ale ucho mutant we observed hundreds of larvae with overinflated endolymphatic sacs (Figure 3A). To qualitatively saturate the distribution of ES inflations and deflations, we acquired 4 time courses where the ES did not deflate. An estimation of sample size needed to dismiss the null hypothesis that the mutant ES inflates and deflates like the wild-type ES would have been on the order of 1 sample, however it is difficult to express this calculation because deflation events were not observed in the mutant. Related time courses where the endolymph was labeled in the mutant were acquired two times (Figure 3E). The experiment where both wild-type and mutant ES cells were sparsely labeled to reveal the presence (wild-type) and absence (mutant) basal projections were performed 15 times for wild-type (Figure 3I) and 9 times for mutant (Figure 3J). Hundreds of embryos were screened to acquire these data because the sparse labeling was random and only ~1/10 embryos had sparse labeling of ES cells. The observations that characterized the apico-basal organization of the ES tissue represent 6-32 acquired images for each condition (Figure 3K).

Appropriate sample sizes were not computed for experiments presented in Figure 4. Data from two ears are presented. More serial section EM was not performed because of time constraints and limited access to the technology. The data represents David Hildebrand’s thesis work that took years of development and analyses. Qualitatively, we reinforce the finding that the relief valve is composed of lamellar junctions with salt and pepper labeling of the ES cells (presented in Figure 3I-J) and observations of patches of brightness that reflect overlapping lamellae (Figure 6F-H). We performed additional analysis of the ES ultrastructure by transmission EM of ultrathin slices on 3 wild-type embryos and 3 mutant embryos (Figure 4H-I)

Appropriate sample sizes were not computed for the experiments presented in Figure 5 because the hypothesis was not generated until we had acquired the higher resolution time courses of the endolymphatic sac. The experiments represented in Figure 5 test the hypothesis that cell thinning and thickening is sometimes correlated, sometimes anti-correlated, and sometimes uncorrelated with ES inflation trajectories. The null hypotheses being dismissed are that cell thickness is always uncorrelated or always correlated with ES inflation trajectories. Spearmen correlation tests were performed for 99 events. Again, because the complex distribution of behaviors we observed, a smaller sample size could have dismissed these null hypotheses, yet we had a desire to qualitatively saturate the range of cell behaviors.

The experiments presented in Figure 6 and 7 qualitatively reinforce the finding of Figure 4 that lamellar junctions are engaged during inflations and open to release pressure. Dismissal of the null hypotheses that lamellar junctions are not present and that lamellar junctions are not dynamic became apparent upon first acquisition of the data. As such, we did not calculate an appropriate sample size. The AO-LLSM data is representative of 4 time-lapse imaging sessions. The myosin and utrophin time-lapses are representative of 4 experiments with wild-type embryos (Figure 6H) and 2 experiments with *lmx1bb* crisper mutants (Figure 6I). The rapid collapse of the dilated mutant ES following puncture is representative of 5 experiments (Figure 6J). The high resolution time-lapse with labeled endolymph (Figure 7E) is representative of 8 such experiments.

Please outline where this information can be found within the submission (e.g., page numbers or figure legends), or explain why this information doesn’t apply to your submission:

**Replicates**

- You should report how often each experiment was performed
- You should include a definition of biological versus technical replication
- The data obtained should be provided and sufficient information should be provided to indicate the number of independent biological and/or technical replicates
- If you encountered any outliers, you should describe how these were handled
- Criteria for exclusion/inclusion of data should be clearly stated
- High-throughput sequence data should be uploaded before submission, with a private link for reviewers provided (these are available from both GEO and ArrayExpress)

Please outline where this information can be found within the submission (e.g., page numbers or figure legends), or explain why this information doesn’t apply to your submission:

All replicate information is now in figure legends.

**Statistical reporting**

- Statistical analysis methods should be described and justified
- Raw data should be presented in figures whenever informative to do so (typically when N per group is less than 10)
- For each experiment, you should identify the statistical tests used, exact values of N, definitions of center, methods of multiple test correction, and dispersion and precision measures (e.g., mean, median, SD, SEM, confidence intervals; and, for the major substantive results, a measure of effect size (e.g., Pearson's r, Cohen's d)
- Report exact p-values wherever possible alongside the summary statistics and 95% confidence intervals. These should be reported for all key questions and not only when the p-value is less than 0.05.

We present the distribution of times between peak inflations for the wild-type in Figure 1G, page 6. The N, mean, and SD are presented on page 5. We did not present a statistical test for the mutant behavior as there was no time between peak inflations. We do present 2 mutant movies.

We used the Mann-Whitney-Wilcoxon method to test the significance of the difference in cell thicknesses observed in Figure 3. The raw measurements are presented in Figure 3G, page 11. The p-value, mean, and SD are presented in the text on page 12.

To test the correlation between trajectories of endolymphatic sac lumen volume and thickness of cells of the endolymphatic sac we used the Spearman rank order correlation test. Information regarding this test can be found in the Results (page 17-18), Figure legend to Figure 5 (page 20), and Materials and methods (page 42). The Spearman test is non-parametric and tests the extent to which data series are monotonic and correlated. The full set of rhos and p-values are presented in Figure 5L.

Please outline where this information can be found within the submission (e.g., page numbers or figure legends), or explain why this information doesn’t apply to your submission:

(For large datasets, or papers with a very large number of statistical tests, you may upload a single table file with tests, Ns, etc., with reference to page numbers in the manuscript.)

**Additional data files (“source data”)**

- We encourage you to upload relevant additional data files, such as numerical data that are represented as a graph in a figure, or as a summary table
- Where provided, these should be in the most useful format, and they can be uploaded as “Source data” files linked to a main figure or table
- Include model definition files including the full list of parameters used
- Include code used for data analysis (e.g., R, MatLab)
- Avoid stating that data files are “available upon request”

Please indicate the figures or tables for which source data files have been provided:

Additional data has been deposited in repositories. Links to Github for the new software developed for this study are on page 42. Links to full source data for the serial EM are on page 43.
